# The Intertwining Between Arthritis and Inborn Errors of Immunity

**DOI:** 10.3390/jcm15093298

**Published:** 2026-04-26

**Authors:** Rita Consolini, Giulia Maestrini, Sarah Abu-Rumeileh, Giorgio Costagliola

**Affiliations:** 1 Section of Clinical and Laboratory Immunology, Pediatric Unit, Department of Clinical and Experimental Medicine, University of Pisa, 56100 Pisa, Italy; 2 Division of Pediatrics, Department of Clinical and Experimental Medicine, University of Pisa, 56100 Pisa, Italy; giulia.mae13@gmail.com (G.M.); sarah.arumeileh@gmail.com (S.A.-R.); 3 Section of Pediatric Hematology and Oncology, Azienda Ospedaliero-Universitaria Pisana, 56100 Pisa, Italy; giorgio.costagliola@ao-pisa.toscana.it

**Keywords:** immunodeficiency, juvenile idiopathic arthritis, common variable immunodeficiency, Wiskott–Aldrich syndrome, autoimmune lymphoproliferative immunodeficiencies (ALPIDs), complement deficiencies, hyper-IgM syndrome, X-linked agammaglobulinemia

## Abstract

Immune dysregulation is being increasingly recognized as a prominent feature of a wide range inborn errors of immunity (IEIs) with different molecular backgrounds. Among the manifestations of immune dysregulation, inflammatory arthritis has emerged as an important yet underrecognized complication that may occur across multiple IEI categories, including humoral immunodeficiencies (such as X-linked agammaglobulinemia, hyper-IgM syndrome, common variable immunodeficiency, and others), complement deficiencies, disorders of immune dysregulation (STAT3 gain of function mutation, CTLA4 and LRBA haploinsufficiency), and combined immunodeficiencies. In some patients, arthritis may represent the first or predominant clinical manifestation, resulting in a diagnostic challenge in the rheumatologic setting. The pathogenesis of arthritis in IEIs reflects different immunological mechanisms, including the defective clearance of immune complexes, dysregulated B- and T-cell responses, impaired regulatory T-cell function, and aberrant cytokine signaling. Clinically, IEI-associated arthritis may mimic classical rheumatologic conditions such as juvenile idiopathic arthritis, rheumatoid arthritis, or other connective tissue diseases, although distinctive immunological and histopathological features are often present. Recognizing arthritis as a potential manifestation of IEIs has important clinical implications. The presence of specific “red flags”, including treatment refractoriness, recurrent infections, or additional signs of immune dysregulation (other autoimmune diseases, atopy, lymphoproliferation, enteropathy), should prompt targeted immunological evaluation. While management often relies on conventional immunosuppressive therapies, advances in the molecular characterization of IEIs are increasingly enabling the use of targeted treatments directed at the underlying pathogenic mechanisms. This paper provides an overview of the current knowledge of arthritis associated with IEIs, highlighting diagnostic challenges, underlying immunopathogenic mechanisms, and emerging therapeutic perspectives.

## 1. Introduction

The term “inborn errors of immunity” (IEIs) has replaced the term “primary immunodeficiencies” (PIDs) as a more encompassing definition, reflecting the recognition of immune dysregulation as the main mechanism underlying the pathogenesis of a large proportion of these immune defects [1]. The International Union of Immunological Societies Expert Committee (IUIS) 2024 update reported 559 IEIs resulting from damaging germline variants in 508 single genes, classified into 10 categories according to the predominant component of the immune system primarily disrupted [1].

Historically, the clinical presentation of IEIs was mainly characterized by increased susceptibility to unusual, severe, or recurrent infections. However, manifestations related to immune dysregulation, including autoimmunity, atopy, autoinflammation, lymphoproliferation, bone marrow failure, and/or malignancy, have been included in their phenotypic picture [2]. These non-infectious manifestations represent the first sign in about 10% of patients [3] with IEIs in the absence of susceptibility to infections. Furthermore, the clinical spectrum of IEIs is gradually expanding [4] due to the evolutionary knowledge of their genetic background and of the divergent molecular mechanisms of variants in the same gene, such as gain of function (GOF), loss of function (LOF), and neomorphic or multimorphic function. The high heterogeneity of the resulting clinical phenotype, often involving multiple organ systems, makes diagnostic work-up particularly challenging. Different specialists may be involved depending on the initial clinical manifestation, often without early collaboration with an experienced immunologist. Early diagnosis is crucial to prevent disease-associated morbidity and mortality; however, most patients with IEIs are not diagnosed until adulthood (mean 49.5 years), with an average delay of approximately 25 years from the onset of disease to diagnosis [5].

Autoimmunity in IEIs has emerged as one of the major manifestations of immune dysregulation. Several immune cell types and molecular signaling pathways have been identified as key pathogenetic mechanisms underlying autoimmunity in these conditions. These include defects in cellular activation, growth, and survival; B-cell dysregulation; abnormal apoptosis; defects in T-cell receptor signaling or immune-mediated clearance; defects in innate immunity; impaired regulatory T-cell differentiation and function; complement defects; and increased type 1 interferon production [6,7,8,9]. This strongly indicates that autoimmunity and immunodeficiency should no longer be considered two mutually exclusive conditions but rather two sides of the same coin, sharing a genetic and/or immune-dysfunctional origin. Cytopenias and rheumatologic diseases are the predominant autoimmune clinical features of IEIs, often associated with a more aggressive clinical course, resistance to conventional treatments, worse prognoses, and increased mortality [10,11].

Rheumatologic diseases are a heterogeneous group of disorders whose pathogenesis involves a complex interplay among genetic and environmental factors and a dysfunctional immune response, leading to a loss of tolerance against self-antigens, finally resulting in organ infiltration by autoreactive lymphocytes and circulating autoantibodies, entailing chronic inflammation and end-organ damage [12,13]. Among the various medical disciplines involved in the IEI work-up and diagnostic process, rheumatology appears the most intriguing, especially in patients with a rheumatic disease as their only or presenting manifestation. The spectrum of rheumatologic diseases associated with IEIs is wide and heterogeneous, with arthritis being one of the most frequent manifestations.

Beyond knowledge of the pathways accounting for immune dysregulation in IEIs and the subsequent rheumatologic manifestations—especially arthritis—the intertwining between IEIs and rheumatology, as focused on in the recent assumption of “blurring borders between immunodeficiencies and rheumatology”, is supported by the identification of risk genes in rheumatic disorders (such as rheumatoid arthritis [RA], juvenile idiopathic arthritis [JIA], and systemic JIA) as disease-causing genes in monogenic IEIs [14]. In the case of rheumatoid arthritis (RA), 20 out of 152 non-MHC susceptibility loci are located in genes linked to IEIs [15,16,17].

The aim of this review is to present the state of the art regarding current knowledge about the various forms of arthritis associated with the most important IEIs, exploring their underlying mechanisms and clinical expression, in order to provide insights into the complexity of the diagnostic process for IEI-associated rheumatologic diseases. Specifically, this paper explores arthritis associated with humoral IEIs, immune dysregulation disorders, syndromic IEIs, and complement deficiencies, while autoinflammatory disorders are not discussed, since their association with arthritis is already characterized in depth.

## 2. IEI-Associated Arthritis: An Overview

### 2.1. Pathogenesis of Arthritis in IEIs

Arthritis in IEIs was originally assumed to be mainly related to the underlying susceptibility to infections, especially by Mycoplasma species, with Ureaplasma urealyticum being one the most important arthritogenic agents and with particular vulnerability in patients with humoral defects [18,19]. Indeed, in a significant number of IEIs, such as phagocyte disorders, infections represent the most frequent etiology of arthritis. More recently, however, non-infectious arthritis has emerged as an important manifestation of immune dysregulation or autoinflammation in several IEIs.

In the last few years, genome-wide association studies (GWAS) have identified a considerable proportion of susceptibility genes for rheumatic disorders that also represent disease-causing genes in monogenic IEIs. Moreover, the pathogenic mechanisms involved in rheumatic diseases have been shown to contribute to IEI-associated immune dysregulation in independent cellular and murine models [20,21]. Overall, the molecular mechanisms involved in the pathogenesis of arthritis in IEIs are multiple and only partly understood. These include altered B-cell homeostasis and tolerance, which is specifically associated with humoral IEIs; a reduced number or function of regulatory T cells (Tregs); impaired lymphocyte apoptosis, such as in diseases of the autoimmune lymphoproliferative immunodeficiency [ALPID] spectrum; and ineffective complement activity. Most of these mechanisms contribute to the pathogenesis of arthritis in most IEI patients; however, in some specific monogenic diseases, a single molecular pathway can be the main factor responsible for the clinical phenotype of immune dysregulation [22,23,24,25,26,27,28,29,30,31,32,33,34,35,36,37,38,39,40,41]. Notably, some of these mechanisms—specifically, those involving B cells—are central players in the development of the major manifestations of several types of arthritis outside the IEI setting, as they are in several IEIs [30,31,32,33,34,35,36,37,38,39,40,41], thus raising the possibility that inflammatory arthritis and IEIs may share common pathophysiological mechanisms, at least in certain arthritis subtypes.

From a pathogenic point of view, it is also important to underline that the histological characteristics of IEI-associated arthritis often differ from those of typical RA or JIA. IEI-associated arthritis can present with synovial hyperplasia and capillary proliferation without lymphocyte or polymorphonuclear infiltration, but with the possible presence of T CD8+ cells. Detailed immunophenotyping (e.g., patterns of arthritis, synovial fluid analysis) is necessary in order to define the characteristics of these patients more precisely.

Other interesting perspectives in this field come from some newly identified monogenic disorders. Specifically, in the last few years, IEIs associated with monogenic systemic lupus erythematosus (e.g., TLR7 GOF mutations) or monogenic inflammatory arthritis (such as biallelic LACC1 loss of function) have been described [1]. As more than 40% of the newly described IEIs have features of immune dysregulation/autoinflammation [1], the better characterization of the pathogenic mechanisms underlying these entities will hopefully lead to an increased understanding also of the pathogenic mechanisms leading to inflammatory arthritis, including outside the IEI setting.

Finally, some relevant pathogenic insights arise from the observation of IEI phenocopies in which arthritis or immune dysregulation is part of the clinical spectrum (e.g., diseases associated with anti-cytokine antibodies). Indeed, these conditions represent, from a research perspective, intriguing models through which to investigate some unexplored aspects of immune dysregulation.

### 2.2. Clinical Features of Arthritis in Patients with IEIs

It is now well known that inflammatory arthritis is the most common rheumatic feature of IEIs, including in pediatric patients [22]. Additionally, through sequencing projects on cohorts of patients with IEIs, arthritis has been recognized as one of the major immune-dysregulatory signs, allowing the diagnosis of an immune defect [23,24].

As a general rule, patients with IEIs may present with monoarticular, oligoarticular, or polyarticular forms, occasionally associated with rheumatoid nodules, and sometimes fulfil diagnostic criteria for JIA or RA [10]. Classic clinical signs of arthritis, such as joint swelling, pain, or functional impairment, may be absent at disease onset. However, initial manifestations such as arthralgia may subsequently progress to overt inflammatory arthritis with joint damage [25].

**Figure 1:**
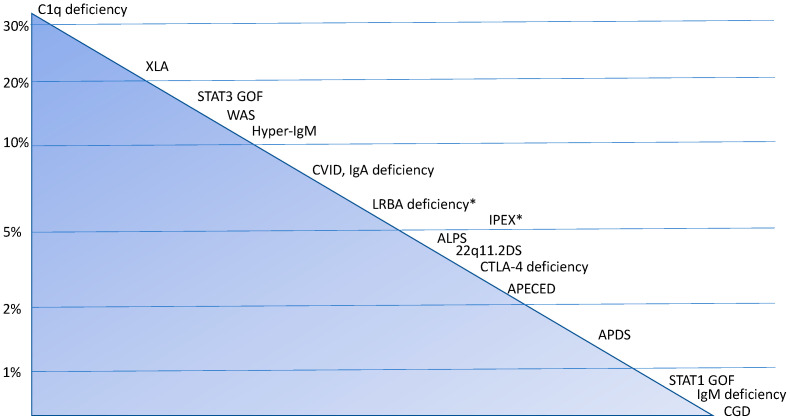
Overview of the main inborn errors of immunity associated with arthritis. Figure legend: * indicates that, for these conditions, the prevalence of arthritis is not separately analyzed, as data include patients with different rheumatic diseases (e.g., arthritis, vasculitis, uveitis). Abbreviations: 22q11.2DS: 22q11.2 deletion syndrome; ALPS: autoimmune lymphoproliferative syndrome; APDS: activated PI3K delta syndrome; APECED: autoimmune polyendocrinopathy–candidiasis–ectodermal dystrophy; CGD: chronic granulomatous disease; IPEX: immune dysregulation, polyendocrinopathy, enteropathy, X-linked syndrome; WAS: Wiskott–Aldrich syndrome; XLA: X-linked agammaglobulinemia.

The most commonly reported associations of IEIs and arthritis, in its various forms, are with complement deficiencies, X-linked agammaglobulinemia (XLA), and common variable immunodeficiency (CVID). However, cohort studies and systematic reviews indicate a variable incidence of arthritis in rare monogenic diseases such as STAT 3 GOF, Wiskott–Aldrich syndrome (WAS), hyper-IgM syndrome (HIGM), immune dysregulation, polyendocrinopathy, enteropathy, X-linked (IPEX) syndrome, autoimmune lymphoproliferative syndrome (ALPS) and related disorders (including CTLA-4 and LRBA haploinsufficiency), autoimmune polyendocrinopathy–candidiasis–ectodermal dystrophy (APECED), activated PI3K delta syndrome (APDS), and others [26,27,28,29,30,31,32,33,34,35,36,37] (Figure 1). In all these conditions—but more frequently in humoral IEIs—diagnosis may be challenging due to the frequent absence of disease-specific autoantibodies [30]; however, while this represents a limitation in conditions such as RA or connective tissue diseases, the diagnosis of JIA remains primarily clinical, with autoantibodies mainly contributing to disease classification and prognostic stratification.

Notably, the prevalence of arthritis shows high variability between different studies. This is partly a result of the retrospective nature of most of these studies and the different criteria used to define arthritis. Indeed, while some studies report only patients with inflammatory arthritis, others include also patients with septic arthritis or arthralgia of unknown origin or classify patients into wider categories according to the presence of rheumatological manifestations (arthritis, vasculitis, and others), without providing specific data on arthritis. Therefore, epidemiological data are still not sufficiently detailed to correctly define the prevalence of arthritis in IEIs or to draw specific age-related insights. Additionally, as previously mentioned, the 2024 update of the IUIS classification includes some new monogenic IEIs in which arthritis can be part of the clinical spectrum [1], thus expanding the clinical and pathogenic spectrum of IEIs associated with arthritis.

## 3. Arthritis in Humoral IEIs

The prevalence of joint manifestations in humoral deficiencies is historically known, ranging from 5% to 40% across different studies in IEI patients [42]. Notably, early reports already suggested that “a combination of arthritis and hypogammaglobulinemia should suggest a PID syndrome” [42]. One of the earliest studies addressing this association was performed by Hansel et al., who identified seven cases of monoarthritis and one case of oligoarthritis among 69 patients with agammaglobulinemia, as well as one case of monoarthritis and seven cases of oligoarthritis among 161 patients with CVID [43].

### 3.1. X-Linked Agammaglobulinemia

XLA is caused by mutations in Bruton’s tyrosine kinase (BTK), encoded by the BTK gene located on the long arm of the X chromosome, which is essential for B-cell receptor-mediated proliferation and survival. The clinical picture is dominated by high susceptibility to severe and recurrent infections, particularly sinopulmonary infections due to encapsulated bacteria [44]. Early reports in XLA patients attributed arthritis primarily to Mycoplasma or other infections rather than to intrinsic immune dysregulation associated with humoral defects. Autoimmunity in this disorder has been linked to aberrant B-cell receptor editing, which confers a survival advantage to B cells with affinity to self-antigens [45] and to improper Toll-like receptor signaling, resulting from the lack of BTK, contributing to defective self-antigen recognition [46]. Although patients with XLA are generally considered to have a low risk of autoimmune or inflammatory disease compared to other humoral defects [47], a higher incidence of autoimmune arthritis in the large joints of the lower extremities has been reported in approximately 20% of patients in some case series [48,49]. Data from a web-based patient survey conducted by the Immune Deficiency Foundation (IDF) in collaboration with the National States Immunodeficiency Network (USIDNET)—the XLA Registry—showed a moderately high frequency of symptoms potentially reflecting an underlying rheumatologic disease, such as painful or swollen joints and arthralgias associated with fatigue, occurring in 34% of patients [49]. This prevalence is comparable to that observed in RA [50], although interpretation is limited by the potential discrepancy between physician-confirmed diagnoses and patient-reported symptoms. A diagnosis of JIA was reached in 16% of patients recruited in the USDINET Registry. Arthritis may be the initial manifestation of XLA, as reported in a case of two siblings initially misdiagnosed as having polyarticular JIA not responsive to conventional therapy before they were diagnosed with XLA [51]. Interestingly, a recent study investigating the frequency of rheumatologic disorders in a pediatric IEI cohort found that approximately half of the patients with rheumatologic disease had JIA in its various forms, with 10% of these cases attributed to XLA; in all cases, arthritis represented the initial clinical manifestation [22].

### 3.2. Common Variable Immunodeficiency (CVID)

CVID is the most frequent symptomatic IEI in both adults and children, presenting with a wide and heterogeneous clinical phenotype [52]. According to the clinical criteria set by the European Society for Immunodeficiencies (ESID), the presence of autoimmune manifestations is one of the key indicators, together with increased susceptibility to infections, granulomatous disease, unexplained polyclonal lymphoproliferation, and an affected family member with an antibody deficiency. The concomitant laboratory evidence of a deficiency in at least two immunoglobulin isotypes—specifically, IgG and IgA and/or IgM (<2SD of the normal levels for age)—is essential to establish a diagnosis after the fourth year of life and after the exclusion of secondary causes of hypogammaglobulinemia [26]. An increased proportion of CD21^low B cells is recognized as an additional diagnostic parameter and has been associated with autoimmune manifestations, particularly autoimmune cytopenias and splenomegaly [9,53]. The genetic background of CVDI is in continuous expansion with increasing knowledge of the genotype–phenotype correlations. Beyond the established monogenetic causes, genes causing diseases with prominent immune dysregulation (CTLA-4 haploinsufficiency and LRBA deficiency) in addition to hypogammaglobulinemia, presenting with a CVID-like phenotype, have been discovered. Furthermore, monogenic defects are more likely to be identified in patients with CVDI with autoimmune complications [54]. The development of autoimmune manifestations in CVID may be due to several factors, including B-cell dysregulation, defective class-switch recombination and receptor editing, the increased survival of autoreactive B cells, the expansion of CD21^low B cells, impaired activation-induced cell death, the reduced clearance of apoptotic cells and immune complexes, and persistent infections [38]. T-cell abnormalities, such as reduced regulatory T cells (Tregs), along with defects in the thymic output of CD4+ T cells, may contribute to the breakdown of self-tolerance and the development of autoimmunity [33]. Moreover, a shared genetic susceptibility has been described between chronic inflammatory arthritis, particularly RA and JIA, and primary antibody deficiencies as mentioned above [14].

Autoimmune manifestations in CVID have a prevalence of 23.2% according to the ESID registry [55], consistent with previously reported estimates of around 20% [56], with similar frequencies reported across different countries [57]. This prevalence exceeds the rate of occurrence in the general population by approximately 7.6-fold and is higher than that of other CVID-related complications (e.g., bronchiectasis, 22.9%; malignancies, 9.35%) [55]. It has also been reported that autoimmunity may occasionally represent the first or only clinical manifestation [58], with autoimmune cytopenia being the most common (affecting over 50% of CVDI patients with autoimmune complications [59,60]). Rheumatologic diseases are also relatively frequent, affecting approximately 6–10% of patients with CVID and occurring more commonly in females [17,25]. Autoimmune arthritis (both RA and JIA) is the most common rheumatological disease, affecting 28% of patients in an Iranian cohort [57]. CVID-associated arthritis can range from mild inflammatory disease to erosive arthritis and may include spondyloarthropathies such as reactive arthritis and inflammatory bowel disease-associated arthritis, as reported in the USDINET cohort [25]. Interestingly, it has been reported that rheumatological autoimmunity coexists with hematological autoimmunity in 3.1% of CVID patients [61], suggesting the need for constant awareness of the appearance of additional signs of autoimmunity along with the monitoring of patients.

### 3.3. Selective IgA Deficiency

Selective IgA deficiency (sIgAD) is the most prevalent IEI, defined by serum IgA levels below 7 mg/dL with normal IgG and IgM levels, in individuals older than four years, after excluding other causes of hypogamaglobulinemia [62]. sIgAD’s clinical phenotype is highly heterogeneous, with some individuals remaining asymptomatic while others develop various comorbidities—mainly infections, primarily affecting the respiratory and gastrointestinal mucosae, and atopic conditions (with a prevalent skin-skewed pattern), due to the lack of the protective role of IgA on mucosal surfaces. Moreover, autoimmune disorders affect approximately one third of patients with sIgAD [63,64]. The clinical phenotype often follows a progressive sequence, with infections being the earliest clinical manifestation, followed significantly by allergic and autoimmune disorders [63,65]. Although autoimmune diseases generally occur later and tend to have a milder course compared with other IEIs [66], clinicians should remain aware of the significant “burden of autoimmunity” affecting sIgAD patients [63,65]. Specifically, arthritis represents a frequent sIgA-associated manifestation, although its prevalence varies according to the ethnic background of the studied population and appears to be more common in Caucasian cohorts [63]. In an extensive literature review, Odineal and Gershwin reported weighted prevalence estimates of 2.7% for RA and 1.97% for JIA among patients with sIgAD, confirming the association between autoimmune arthritis and this immunodeficiency [36]. Interestingly, it has been reported that there is a significantly greater prevalence of RA in sIgAD patients than in the general population [67], whereas results regarding the association with JIA remain inconsistent [63].

sIgAD represents an interesting model through which to explore the pathogenesis of autoimmunity in IEIs, although the serologic and B-cell-intrinsic pathways underlying the immune dysregulation of sIgA deficiency remain poorly defined. Strong associations between certain HLA haplotypes have been observed in European cohorts, some of which overlap with those associated with autoimmune diseases [68,69]; however, these findings are not consistently reproduced across different populations worldwide [70]. Furthermore, no single mutation has yet been found to explain the correlation of sIgAD with any specific autoimmune disease or autoimmunity in general, suggesting that additional mechanisms must contribute in order for any individual to develop autoimmune manifestations [36]. Abolhassani et al. observed that patients with sIgAD complicated by autoimmunity had fewer regulatory T cells, as well as CD27+ IgD− memory B cells, compared to sIgAD patients without autoimmunity [63]. Cellular phenotyping in sIgAD has demonstrated a defect in transitional B cells enhanced by TLR9 stimulation, pointing towards an early B-cell defect linked to T-cell-independent B-cell responses [71] and impaired IL-21-driven STAT3 B-cell activation [72], leading to B-cell dysregulation. Notably, a recent investigation of the serological and transcriptomic profiles of a cohort of adult sIgAD cells showed an elevated systemic IL18-centered soluble signature coexisting with reduced transcriptional expression of the IL-18 receptor components, which has been associated with self-reactive antibody responses [73], with consequently altered signaling programs in B cells [65]. It is known that patients with sIgAD exhibit elevated levels of IgG and IgM, along with a higher prevalence of autoantibodies and autoimmune disorders without clinical manifestation and no links to the affected underlying immunological pathways [65,74]. Therefore, based on these immunological peculiarities, Lemarquis et al. suggest that the endotype (IL-18-IL-18R axis) observed in the abovementioned sIgAD cohort of patients aligns with ANA/ENA positivity and an IgG-skewed class-switch profile, providing a blood-measurable framework for understanding immune dysregulation beyond the defining IgA deficiency [65].

### 3.4. Hyper-IgM Syndrome

Hyperimmunoglobulin M (HIGM) syndrome encompasses a group of disorders characterized by normal-to-elevated levels of serum IgM and significantly reduced or undetectable levels of IgG, IgA, and IgE [75]. This defect results from an impairment in the CD40L-CD40 pathway, which plays a pivotal role in B-cell class-switch recombination and somatic hypermutation. The X-linked variant is the most common subtype (65–70%) of HIGM syndrome, although rarer autosomal recessive variants have been identified. Mutations in CD40L are the most frequent among the seven known genetic defects associated with this condition [76]. Patients with HIGM are at risk for recurring bacterial and fungal infections, which largely impact the respiratory and gastrointestinal systems. In addition to susceptibility to infections, HIGM patients are prone to developing autoimmune diseases with variable presentation [77] according to the underlying genetic defect [78]. Autoimmunity in HIGM could be explained by the presence of IgM autoantibodies, decreased peripheral B-cell tolerance [79], the impaired development of Treg cells, and increased levels of BAFF (a potent B-cell survival factor [80]).

There are no recent studies on the clinical phenotype of HIGM. Historical analyses reported an incidence of 11% of seronegative arthritis [81] and one case of a form of polyarticular arthritis complicated by subcutaneous nodules and periarticular cysts [82], with a recent report of polyarticular JIA in a 4-year-old patient [83], confirming the aggressive modality of presentation.

## 4. Arthritis in Complement Deficiencies

The complement system operates through three distinct pathways, namely the classical, alternative, and lectin pathways, which ultimately converge with the activation of C3 and initiate a cascade leading to the formation of the membrane attack complex (MAC)—a key mechanism for pathogen lysis [84]. Beyond its role in host defense against pathogens, the complement system is involved in several critical aspects of immune regulation, including the clearance of immune complexes, the release of anaphylatoxins with pro-inflammatory activity, and others [85]. Consequently, congenital deficiencies in proteins involved in the complement system are associated with a wide spectrum of manifestations, ranging from increased susceptibility to infections (especially by encapsulated pathogens like pneumococcal and meningococcal disease) to autoimmune diseases, including connective tissue diseases (systemic lupus erythematosus [SLE]-like pictures), nephritis, and arthritis [85,86]. Specifically, autoimmune manifestations, often occurring in association with infections, are most frequently observed in patients with deficiencies in early complement components, such as C1q and C4. In contrast, deficiencies affecting components involved in MAC formation (C5–C8) are primarily associated with increased susceptibility to infections [85]. Other complement defects linked to autoimmunity, although less frequently, include deficiencies in C2 and C3. Regarding arthritis, studies report an incidence of up to 38% in patients with C1q deficiency [87], often in the context of a broad autoimmune phenotype with SLE-like features. Although the prevalence of arthritis in patients with C4 deficiency is not defined in epidemiological studies, its association with an articular phenotype has been described in several reports. Moreover, partial C4 deficiency is reported as a risk factor for the development of JIA, especially in patients with a history of severe infections [88], while studies focusing on RA have found a relevant proportion of patients with C4B deficiency [89].

The pathogenesis of arthritis in complement deficiency is complex and involves multiple interconnected molecular mechanisms. Indeed, complement deficiency results in the impaired clearance of immune complexes, leading to the persistence of autoantigens and potentially promoting increased autoantibody production [85,90].

From a clinical point of view, an underlying complement deficiency should be suspected when arthritis or an early SLE-like phenotype coexists with increased susceptibility to severe infections. As will be further discussed, the isolated analysis of serum complement levels (C3 and C4) is often not enough to rule out a complement deficiency, highlighting the need for functional assays specifically designed to assess the activity of the different complement pathways.

## 5. Arthritis in Immune Dysregulation Disorders

### 5.1. Arthritis in Disorders of Regulatory T Cells

Disorders affecting Tregs encompass a wide spectrum of conditions, which include IPEX, STAT1 and STAT3 GOF mutations, CTLA4 and LRBA haploinsufficiency, and others. In most Treg disorders (Tregopathies), immune dysregulation represents the prominent disease feature, being expressed with autoimmunity (cytopenia, endocrinopathy, rheumatic diseases), enteropathy, and atopy, eventually accompanied by lymphoproliferation [91].

The most widely known Tregopathy is IPEX syndrome, caused by mutations impairing the FOXP3 transcription factor and, therefore, the differentiation of Tregs [92]. IPEX is typically characterized by a triad of eczema, autoimmune endocrinopathy, and enteropathy, but other autoimmune diseases are common, with arthritis or vasculitis being reported in 5–10% of patients [31].

A higher prevalence of arthritis among Tregopathies has been reported in patients with STAT3 GOF mutations (18%) [27], caused by the uncontrolled activation of the JAK/STAT molecular pathway, resulting in increased cytokine production and widespread immune dysregulation [93]. On the other hand, the prevalence of arthritis in patients with CTLA4 or LRBA deficiency is less well characterized. Indeed, in these two pathogenically related conditions impairing the expression of CTLA-4 on Treg surfaces (which is essential in inducing anergy in self-reactive lymphocytes) [94,95], the largest available review studies have pointed out only the cumulative incidence of rheumatic conditions (arthritis, vasculitis, and uveitis) rather than arthritis alone [26].

Notably, in STAT-associated diseases, as well as CTLA4 and LRBA deficiency, beyond the IPEX-associated signs, patients often experience hematological involvement, including autoimmune cytopenia, lymphoproliferation, and an increased risk of lymphoma [95], with a clinical picture that can also fulfil the diagnostic criteria for autoimmune lymphoproliferative immunodeficiency (ALPID) [96], thus resulting in extremely complex clinical pictures.

### 5.2. Arthritis in Other Diseases of the ALPID Spectrum

Beyond Tregopathies, arthritis can be a feature of several other immune dysregulation disorders included in the autoimmune lymphoproliferative immunodeficiency (ALPID) spectrum, such as autoimmune lymphoproliferative syndrome (ALPS) and activated PI3Kδ syndrome (APDS). ALPS is caused by mutations impairing FAS-mediated lymphocyte apoptosis and is commonly characterized by autoimmune cytopenia, polyclonal lymphoproliferation, and an increased risk of lymphoma [97], although other autoimmune manifestations have been described [98]. In this condition, arthritis is reported in about 4% of patients [34]. Typical laboratory features of ALPS include the elevation of double-negative T cells, vitamin B12, soluble FAS ligand, IL-10, and IL-18, as well as an impaired apoptotic function assay [99].

APDS, although historically classified within humoral IEIs, often presents with an ALPS-like clinical phenotype, which can also be accompanied by increased susceptibility to sinopulmonary and viral infections [100,101]. In APDS, arthritis is not a common feature, being described in less than 5% of cases. However, its pathogenesis deserves special consideration. Indeed, the clinical and immunological picture of APDS relies on the uncontrolled activation of PI3K-dependent molecular pathways, finally resulting in a tendency towards lymphocyte proliferation, differentiation, and senescence [101]. As specific PI3K inhibitors (especially leniolisib) are available in clinical practice, when approaching a patient with a suspected IEI and immune dysregulation, including arthritis, ruling out APDS is of pivotal importance [102].

## 6. Arthritis in Wiskott–Aldrich Syndrome and Combined Immunodeficiencies

Wiskott–Aldrich syndrome (WAS) is a rare X-linked disease caused by mutations impairing the function of WAS-associated protein (WASP) and featuring a highly variable spectrum of disease severity [103,104]. Indeed, according to the clinical severity, it is possible to distinguish classical WAS—characterized by the classic combination of small-sized thrombocytopenia, immunodeficiency, and eczema—from X-linked thrombocytopenia, in which the impairment of the immune system is less prominent [105]. Among these two entities, the clinical spectrum and variability of WAS are considerable, involving also a considerable rate of autoimmune manifestations.

As WASP is implicated in cytoskeleton remodeling, it is therefore involved in various aspects of lymphocyte-to-lymphocyte interactions (immunological synapses), motility, adhesion, and effector function. Therefore, the pathogenesis of autoimmunity in WAS, which is not fully understood, involves multiple mechanisms, including the impaired function of Tregs, the defective homing and function of B cells, ineffective pathogen clearance, the uncontrolled activation of TLR-dependent pathways, and defective apoptosis [30,106]. These mechanisms may contribute differently to the pathogenesis of the various autoimmune manifestations observed in WAS, potentially explaining the wide clinical variability of the disease. Overall, autoimmune manifestations have been reported in 26–72% of patients with WAS, reflecting considerable variability across different studies [29,30,106]. The most commonly reported autoimmune manifestations include autoimmune cytopenia, vasculitis, arthritis, enteropathy, renal disease, and cutaneous autoimmunity.

The incidence of arthritis in WAS also varies dramatically between different studies [106], being reported with frequencies ranging from 1–3% to 21–29% [107,108], with this divergence likely depending on the duration of follow-up and the inclusion criteria (e.g., WAS only or WAS and XLT). Notably, some of the studies reporting a higher prevalence of arthritis also included transient presentations, while the frequency of chronic arthritis is generally lower than 15% [107].

Data about the incidence of arthritis (especially in its chronic presentation) are limited, as well as for other syndromic and combined immunodeficiencies. A paradigmatic example is represented by 22q11.2 deletion syndrome (22q.11.2DS), in which a recent study by Liebling et al. analyzed the features of chronic inflammatory arthritis in 30 patients [109]. These findings are particularly relevant since they highlight that IEI-associated arthritis, although frequently classified within nosological categories (e.g., JIA), may have significant features and a potentially aggressive disease course. Consequently, dedicated studies are needed to better define the disease characteristics and optimize clinical management and follow-up in this patient population.

## 7. Clinical Implications

The current understanding of autoimmune diseases and IEIs, as two sides of the same coin, means that the appearance of a disorder from “one side” should prompt alertness to possible manifestations of “the other side” [110].

Moreover, due to the overlapping of the genetic background and clinical phenotype of IEIs and rheumatic disorders, as well as the interference of multiple autoimmune manifestations and/or infections in the trajectory of the IEI clinical picture, the diagnostic work-up and clinical care of patients with arthritis primarily occurring in an immunological or rheumatological setting currently represents a significant challenge for clinicians.

### 7.1. Diagnosing Arthritis in Patients with IEIs: Red Flags in the Immunological Setting

Patients with IEIs are at an increased risk for different rheumatologic manifestations, including vasculitis, early-onset connective tissue diseases, granulomatous diseases, organ-specific autoimmunity, and arthritis [10,11]. Therefore, at the time of diagnosis and during periodical clinical assessment, clinicians should investigate the presence of specific signs, including arthralgia, myalgia, xerostomia, xeropthalmia, aphthosis, photosensitivity, and cutaneous findings (e.g., vasculitis, Raynaud’s phenomenon). The presence of rheumatic diseases, particularly arthritis, should be particularly assessed in IEI patients presenting with prominent features of immune dysregulation [24] and/ or with an associated higher incidence of arthritis (complement deficiency, WAS, XLA, and others). Multiple joints, such as the knees, ankles, and hands, can be affected, especially in children, sometimes without clear symptoms of arthritis, like swelling, pain, or functional impairment. This suggests that a complete joint examination must be included in the IEI patient’s general physical examination, preferably performed by a rheumatologist. The histological characteristics can differ from those of typical RA or JIA, as mentioned above, and diagnosis is often complicated by the absence of autoantibodies in the case of humoral defects [30].

Despite several efforts to provide a correlation between laboratory findings and the clinical phenotypes of patients with IEIs, currently, there is a lack of immunological predictors of arthritis. As previously discussed, it is known that patients with CVID and increased CD-21-low B cells have a higher risk of developing autoimmune diseases [111]. In this condition, the analysis of B-memory subsets has been studied in an attempt to provide a correlation with the clinical phenotype of autoimmunity, granulomatous disease, and lymphoproliferation, albeit with inconclusive results [112]. Similarly, extended T-cell phenotyping has been explored to search for predictors of immune dysregulation in 22q11.2DS. From these studies, it has emerged that T-cell lymphopenia and skewing towards memory T-cell populations, with reduced levels of naïve T cells and recent thymic emigrants (RTEs), are more frequently associated with autoimmunity [113,114,115,116,117]. Of note, none of the mentioned previous studies had a specific focus on the immunophenotyping markers associated with arthritis. Therefore, the characterization of this aspect in greater depth is warranted to optimize the early identification of arthritis in patients with a known diagnosis of IEI. In this scenario, the identification of new monogenic disorders will hopefully help in better determining the specific risk of arthritis in distinct clinical entities.

### 7.2. Diagnosing IEIs in Patients Presenting with Arthritis: Red Flags in the Rheumatologic Setting and a Proposed Approach

#### 7.2.1. Identification of Red Flags for IEIs in the Rheumatology Setting

**Figure 2:**
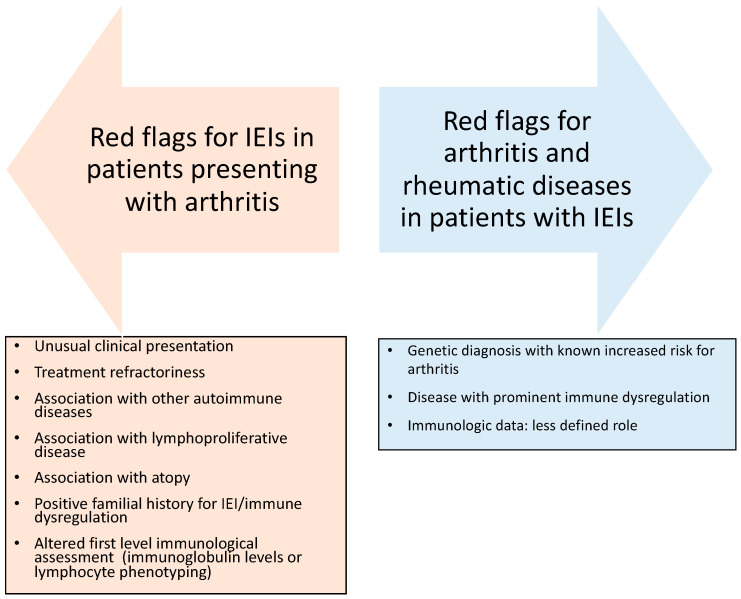
Red flags regarding suspicion of inborn errors of immunity in patients with arthritis and for the early recognition of arthritis in patients with inborn errors of immunity.

Increasing knowledge of the wide clinical spectrum of IEIs is leading to greater awareness of the risk of arthritis in patients with IEIs. In studies conducted in rheumatologic patients, the prevalence of IEIs was estimated to be 14–22%, and the presence of IEIs was associated with a worse prognosis and a more severe disease outcome [10,22]. Furthermore, the prevalence of rheumatic disease in IEIs, and especially the proportion of patients with a rheumatic disease as their only manifestation, may be underestimated, since most patients undergo IEI genetic testing for their immunodeficiency (i.e., for recurrent infections) [8]. Arthritis can represent the leading or the only sign of several IEIs, thus being able to hide or complicate the identification of an underlying IEI, manifesting as a typical rheumatic disorder (RA, JIA, psoriatic arthritis, or others). In these cases, arthritis arises before the onset of clinical and laboratory features of immunodeficiency and may develop during treatment with disease-modifying antirheumatic drugs (DMARDs) and steroids [14]. Although challenging, early IEI identification in a patient presenting with arthritis can offer opportunities to improve their clinical care, preventing disabling complications. Guidelines addressing this specific diagnostic challenge are lacking. However, given the peculiarity of the molecular background and clinical presentation of IEIs with rheumatological involvement, it is worth underlining that specific warning signs, when evidenced in patients with arthritis, should always alert the clinician to the possibility of an underlying IEI. The role of a positive familial history of immune dysregulation or immunodeficiency is extremely important given the variable expressivity that is common among disorders of immune dysregulation, and this must be considered a definite “red flag” for an underlying IEI [8]. On the other hand, data about the age at presentation do not allow the consideration of an early age at the onset of arthritis as a warning sign of IEI, being mostly limited to cohort studies performed on specific monogenic disorders (e.g., LRBA deficiency) [118]. The unusual, aggressive clinical course—and particularly the treatment refractoriness—of arthritis is also highly suggestive of an underlying IEI. The coexistence or a previous history of infectious susceptibility, especially in patients without predisposing factors, represents another element of diagnostic suspicion [3], as indicated by the Jeffrey Modell Foundation’s (JMF) warning signs [119]. Moreover, the complex clinical spectrum of IEIs focuses on the non-infectious phenotype, highlighting the need to extend the 10 warning signs of the JMF with the inclusion of immune-dysregulatory signs and malignancies [2]. A retrospective analysis of a wide cohort of IEIs, aiming to determine and compare the utility of the JFM warning signs with its extended version in IEI diagnosis, showed that the strongest predictors of severe IEIs were hemato-oncologic disorders, a positive family history, and autoimmunity [120]. From the evidence described in this paper, it is reasonable to consider a potential IEI in all patients with arthritis showing an association with other features of immune dysregulation (autoimmune diseases, LPD, atopy), infectious susceptibility, and a positive familial history of IEIs, immune dysregulation, or recurrent/severe infections. Moreover, as a general rule, patients with unusual clinical presentations or marked treatment refractoriness should undergo in-depth immunological evaluation. Notably, as a first-level immunological assessment, including serum immunoglobulin levels and standard lymphocyte subpopulations can guide the diagnosis of an IEI, and it is reasonable to perform this in all patients with arthritis—or at least in those showing treatment-refractory disease (Figure 2).

#### 7.2.2. Proposed Diagnostic Approach for Patients with Arthritis and Suspected IEIs

**Figure 3:**
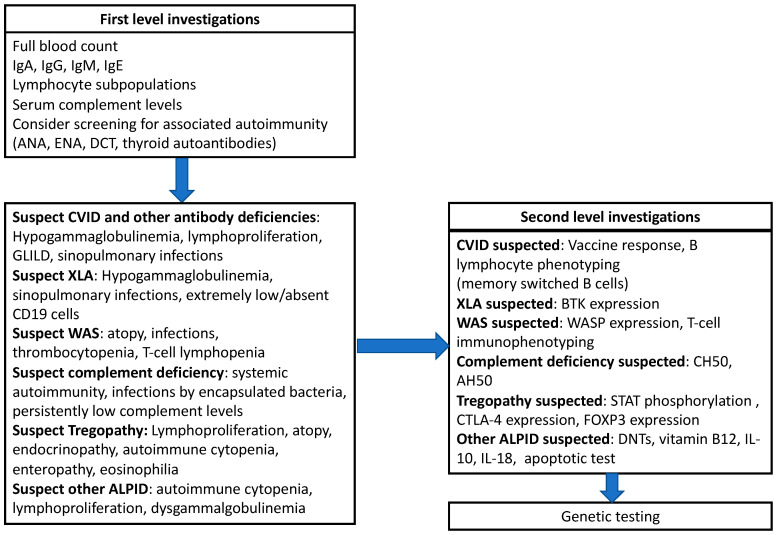
Proposal of diagnostic approach for patients with arthritis and suspected inborn errors of immunity.

Beyond the identification of patients at risk for IEIs, an accurate clinical and first-level assessment can, in selected cases, also help in obtaining a specific diagnosis. However, as many IEIs associated with arthritis share overlapping clinical and laboratory features, differential diagnosis remains challenging in most cases. Beyond first-level analysis (full blood count, immunoglobulin levels, lymphocyte subpopulations), patients with at least one red flag for an IEI should receive a second-level immunological assessment, including targeted investigations, based on the clinical suspicion. Specifically, in patients with hypogammaglobulinemia affecting two or more isotypes, an analysis of the vaccine response and the quantification of switched-memory B cells can be performed to rule out a diagnosis of CVID [121], while extended T-cell phenotyping (with a focus on RTEs and naïve T cells) is of significant help in patients with a suspected combined immunodeficiency. In cases of a suspected complement deficiency, apart from the determination of serum complement proteins, the assessment of complement activity is recommended. As the complement deficiencies that are commonly linked to arthritis involve the classical pathway (C1, C2, and C4), the analysis of CH50 is the diagnostic gold standard in this setting [85]. Integration with AH50 may help to identify other complement deficiencies involving early components or factors implicated in MAC formation, although this approach is more commonly performed in patients with a predominantly infectious phenotype. Additional investigations can be performed to rule out specific diagnoses, such as FOXP3 expression in suspected IPEX, CD40-CD40L in HIGMs, and BTK in suspected XLA, among others. Finally, genetic analysis can be performed through single-gene analysis in the case of a specific suspicion—or, more often, the use of targeted next-generation sequencing or whole-exome sequencing panels is used. Figure 3 summarizes the proposed critical approach to patients with arthritis and suspected IEIs.

## 8. Treating Arthritis in Patients with IEIs: The Role of Targeted Therapies

Currently, there are no specific guidelines for the treatment of arthritis in individuals with a suspected or diagnosed IEI. Therefore, these patients commonly receive the same therapeutic approach used in the non-IEI setting. From a clinical point of view, a multidisciplinary approach involving an immunologist can potentially improve patient outcomes. Indeed, in this population, the treatment of arthritis should necessarily be accompanied by supportive immunologic care including infectious surveillance, the optimization of vaccination strategies, and, when indicated, antibiotic prophylaxis and/or immunoglobulin replacement therapy.

Notably, considering the high burden of immune dysregulation in individuals with IEIs, arthritis in this population may follow a more severe and treatment-refractory course in a significant proportion of cases, as previously discussed. Moreover, some potential concerns in the therapeutic approach to this category of patients are historically represented by the use of immunosuppressive treatments, including biologic agents, in patients with already compromised immune function and increased infection risks. However, literature data—although extremely limited and derived from isolated case reports or case series—seem to indicate the feasibility of biologics in patients with arthritis and IEIs [122]. Beyond conventional treatments (including DMARDs), in patients with arthritis and IEIs, obtaining a molecular diagnosis is pivotal, since, in some specific cases, therapies directly targeting the underlying molecular defect are available [123]. This is the case for STAT-associated disorders, in which the use of JAK inhibitors has demonstrated clinical efficacy [124]. Similarly, in patients with CTLA-4 and LRBA haploinsufficiency, the use of the CTLA-4 fusion protein abatacept is correlated with a high response rate [125], as well as the administration of leniolisib in patients with APDS, although the latter has been mostly studied in patients with a phenotype of autoimmune cytopenia and lymphoproliferation [126]. As most data about the effectiveness and safety of many targeted treatments are derived from studies not specifically investigating patients with arthritis, further research is needed to accurately assess the role of these therapies in this specific setting.

Finally, as research in the field of IEIs associated with immune dysregulation is rapidly progressing, preclinical studies are pointing out some candidates for further targeted therapies. These developments will hopefully translate into improved and more personalized therapeutic strategies for this complex group of patients.

## 9. Concluding Remarks and Future Directions

Increasing evidence indicates that inflammatory arthritis may occur across a wide spectrum of IEIs, ranging from humoral immunodeficiencies to disorders of immune dysregulation, combined immunodeficiencies, and complement deficiencies. Despite this growing recognition, important gaps remain in the current literature. In particular, data regarding the prevalence of arthritis across different IEIs, as well as the detailed characterization of its clinical patterns (monoarticular, oligoarticular, or polyarticular) and its potential classification within established rheumatologic entities (e.g., JIA), remain limited in many available studies.

Furthermore, the continuous discovery of new monogenic IEIs is progressively expanding our knowledge about the mechanisms underlying immune dysregulation but also adds complexity to the interpretation of clinical data related to arthritis in these disorders. For these reasons, dedicated studies are needed to better define the clinical and immunological characteristics of arthritis associated with IEIs. Improved knowledge in this field may facilitate the earlier recognition of underlying IEIs among the large population of patients presenting with arthritis and support the identification of a molecular diagnosis. This final aspect is of specific relevance given the possibility—for specific IEIs—to adopt therapies that specifically target the underlying molecular defect, which may ultimately lead to improved clinical outcomes and more personalized approaches to patient care.

## Data Availability

Not applicable.

## Abbreviations

The following abbreviations are used in this manuscript:

ALPS	Autoimmune lymphoproliferative syndrome
APDS	Activated PI3K delta syndrome
APECED	Autoimmune polyendocrinopathy–candidiasis–ectodermal dystrophy
CVID	Common variable immunodeficiency
DMARDS	Disease-modifying antirheumatic drugs
ESID	European Society for Immunodeficiencies
GOF	Gain of function
GWAS	Genome-wide association studies
HIGM	Hyper-IgM
IEI	Inborn errors of immunity
IPEX	Immune dysregulation, polyendocrinopathy, enteropathy, X-linked
IUIS	International Union of Immunological Societies Expert Committee
JIA	Juvenile idiopathic arthritis
JMF	Jeffrey Modell Foundation
LOF	Loss of function
LPD	Lymphoproliferative disease
MAC	Membrane attack complex
PIDs	Primary immunodeficiencies
RA	Rheumatoid arthritis
sIgAD	Selective IgA deficiency
SLE	Systemic lupus erythematosus
Tregs	Regulatory T cells
WAS	Wiskott–Aldrich syndrome
WASP	WAS-associated protein
XLA	X-linked agammaglobulinemia
